# Draft Genome Sequence of Mariprofundus micogutta Strain ET2

**DOI:** 10.1128/genomeA.00342-18

**Published:** 2018-05-17

**Authors:** Hiroko Makita, Shinro Nishi, Yoshihiro Takaki, Emiko Tanaka, Takuro Nunoura, Satoshi Mitsunobu, Ken Takai

**Affiliations:** a Japan Agency for Marine-Earth Science and Technology (JAMSTEC), Yokosuka, Kanagawa, Japan; b Department of Applied Chemistry, Kanagawa Institute of Technology, Atsugi, Kanagawa, Japan; c Department of Environmental Conservation, Graduate School of Agriculture, Ehime University, Tarumi, Matsuyama, Japan

## Abstract

Mariprofundus micogutta strain ET2 was isolated in 2014 from a deep-sea hydrothermal field on the Bayonnaise Knoll of the Izu-Ogasawara arc. Here, we report its draft genome, which comprises 2,497,805 bp and contains 2,417 predicted coding sequences.

## GENOME ANNOUNCEMENT

Mariprofundus micogutta ET2 (=KCTC15556^T^=JCM30585^T^) is a microaerophilic and chemolithoautotrophic mesophile that grows by oxidizing ferrous iron. The stalk-forming type strain M. ferrooxydans PV-1 and the non-stalk-forming strain M. ferrooxydans JV-1 were the first reported isolates belonging to the genus Mariprofundus (1). Since that initial isolation, strains belonging to the class “Candidatus (*Ca*.) Zetaproteobacteria” have been discovered throughout the Pacific and Atlantic oceans, mainly from deep-sea hydrothermal fields (2). Recently, M. ferrooxydans strains were successfully isolated from a salt marsh of the Great Salt Bay, Newcastle, Maine (3), and the Spillway Area of the Loihi Seamount (4). Here, we report the genetic features of M. micogutta strain ET2, which were determined using draft genome sequencing.

Strain ET2 was isolated from sediment of a 772-m-deep hydrothermal field on the Bayonnaise Knoll, Izu-Ogasawara Arc. A pure culture was obtained by a dilution-to-extinction series with artificial seawater (ASW) medium, as previously described (5). Strain ET2 was grown in ASW medium at 25°C with filament formation. Genomic DNA was extracted from cell pellets using a PowerMax Soil DNA isolation kit (Mo Bio, Carlsbad, CA, USA). A sequencing library was prepared with a Nextera DNA prep kit (Illumina, Inc., San Diego, CA, USA) and subsequently sequenced using an Illumina MiSeq version 3 reagent kit (600 cycles) with 300-bp paired-end reads on the Illumina MiSeq platform. A total of 17.6 million reads were produced, yielding 7.5 Gb of data. Read quality was assessed with FastQC (https://www.bioinformatics.babraham.ac.uk/projects/fastqc). Quality-controlled reads were used to assemble the draft genome with SPAdes version 3.5.0 (6), resulting in 59 contigs with an *N*_50_ value of 105,204 bp (the largest contig was 249,769 bp); the assembled draft genome was 2,497,805 bp in size with a G+C content of 48.75%. Annotation was carried out using Prokka version 1.11 (7), with 2,417 coding sequences (CDSs), 6 rRNAs, and 49 tRNAs being identified. Average nucleotide identity (ANI) comparisons with other M. ferrooxydans genomes, including strains PV-1 and M34, revealed ANIs of 95.89% and 95.8%, respectively. This confirms the placement of M. micogutta strain ET2 in the genus Mariprofundus of the class “*Ca*. Zetaproteobacteria.” Similar to strains PV-1 and M34, strain ET2 encodes genes for growth by chemoautotrophy and motility via a flagellum, although motility was not observed in laboratory experiments and observations (5). A complete set of CDSs for carrying out carbon fixation using the Calvin-Benson-Bassham cycle was identified. Unlike other members of the class “*Ca*. Zetaproteobacteria”, M. micogutta strain ET2 has both *aa3*- and *cbb3*-type oxidase genes. The genome includes eight chemotaxis (*che*) genes and four methyl-accepting chemotaxis proteins, which were previously shown to play important roles in adaptation to deep-sea vent environments (8). Moreover, since the cobalt-zinc-cadmium resistance protein (*czc*) is present, it is considered to exhibit heavy-metal tolerance (9). The outer membrane cytochrome Cyc2 (10), a possible key gene for catalyzing iron oxidation, was detected. Genes for the putative outer membrane Fe oxidase MtoA (11, 12) were not found in the ET2 genome, like other Mariprofundus species.

### Accession number(s).

The whole-genome shotgun project reported here was deposited at DDBJ/EMBL/GenBank under the accession number BDFD00000000, and the 59 contigs generated were deposited under the accession numbers BDFD01000001 to BDFD01000059.
